# InVAErt networks for amortized inference and identifiability analysis of lumped‐parameter haemodynamic models

**DOI:** 10.1098/rsta.2024.0215

**Published:** 2025-04-02

**Authors:** Guoxiang Grayson Tong, Carlos A. Sing-Long, Daniele E. Schiavazzi

**Affiliations:** ^1^ Department of Applied and Computational Mathematics and Statistics, University of Notre Dame College of Science, Notre Dame, IN 46556, USA; ^2^ Institute of Mathematical and Computational Engineering, Pontificia Universidad Católica de Chile, Santiago, Chile

**Keywords:** identifiability analysis, computational haemodynamics, amortized inference, inverse problems, electronic health records

## Abstract

Estimation of cardiovascular model parameters from electronic health records (EHRs) poses a significant challenge primarily due to lack of identifiability. Structural non-identifiability arises when a manifold in the space of parameters is mapped to a common output, while practical non-identifiability can result due to limited data, model misspecification or noise corruption. To address the resulting ill-posed inverse problem, optimization-based or Bayesian inference approaches typically use regularization, thereby limiting the possibility of discovering multiple solutions. In this study, we use inVAErt networks, a neural network-based, data-driven framework for enhanced digital twin analysis of stiff dynamical systems. We demonstrate the flexibility and effectiveness of inVAErt networks in the context of physiological inversion of a six-compartment lumped‐parameter haemodynamic model from synthetic data to real data with missing components.

This article is part of the theme issue ‘Uncertainty quantification for healthcare and biological systems (Part 2)’.

## 1. Introduction

Lumped‐parameter (also known as zero-dimensional or 0D) haemodynamic models provide computationally inexpensive representations of the human cardiovascular system and are widely used either as low-fidelity surrogates or to provide closed-form boundary conditions in three-dimensional cardiovascular simulations [1–4]. Such representations model the evolution of bulk haemodynamic quantities of interest (QoI), simulating the time evolution of organ-level pressure, volume and flow [1,5], disregarding any spatial dependence. They can be represented as circuit models arranged by compartments and formulated as systems of ordinary differential equations (ODEs) the solutions of which are computed using numerical solvers.

Despite their simplicity, working with 0D models can be challenging. First, these models are often described by stiff ODE systems [6,7], where fast and slow dynamics with wildly different response rates coexist in the same system, and therefore, benefit from a careful selection of the numerical algorithm and time‐step size. Second, due to their reduced computational cost, these systems tend to be overparametrized, which typically induces structural non-identifiability [8,9] in inverse problems aiming to assess cardiovascular function from the observed physiological response [7,10–13]. In addition, practical non-identifiability may also manifest as a result of poor data quality, model misspecification and aleatoric uncertainty. Lack of identifiability can induce solution non-uniqueness, excessive uncertainty and convergence failure in numerical algorithms [8,14].

Parameter estimation, sensitivity and identifiability analysis of lumped‐parameter haemodynamic models are extensively studied in the literature. An incomplete list of contributions includes the analysis of structural identifiability in a six-compartment 0D cardiovascular model in [13], which shows that global identifiability can be achieved by adjusting the clinical targets and modifying the state equations accordingly. The method of profile likelihood [9] is applied to investigate practical non-identifiability in a three-compartment haemodynamic model [12], where likelihood-based confidence intervals indicate whether a parameter is practically identifiable. In [2,7,10,11], Bayesian inference and an eigenanalysis of the Fisher information matrix are used jointly for model calibration, uncertainty quantification and local identifiability analysis in the context of lumped‐parameter haemodynamics. Finally, a recent neural network-based approach for simulation-based inference ([15]) has been explored in [16], where neural posterior estimation is applied to a one-dimensional whole-body cardiovascular model.

In this article, we perform *model synthesis* for a six-compartment lumped‐parameter haemodynamic model [6] using the recently proposed framework of inVAErt networks [17]. InVAErt networks are designed to learn *enhanced* digital twin representations, which extend simple emulation with the ability to solve amortized inverse problems and to assess identifiability. To handle ill-posed inverse problems with multiple solutions, a variational encoder learns an input-dependent latent space, which is used to restore bijectivity in the input-to-output map. Decoding the combination of latent space realizations and observations allows one to obtain entire manifolds of solutions. This is in sharp contrast with classical approaches based on regularization (e.g. [18]), that enforce uniqueness through augmented penalized objectives, preventing the possibility of characterizing solution multiplicity.

Bayesian methods can potentially find multiple solutions through posterior analysis. However, they may encounter challenges due to their heavy reliance on prior distributions, the efficiency of Markov chain Monte Carlo-type samplers in high-dimensional problems and poor mixing in non-identifiable models [8,14].

While previous literature on inVAErt networks related to numerical examples from linear and nonlinear maps, dynamical systems and spatio-temporal PDEs, this article demonstrates extensions to applications involving noisy and missing data. Noise is injected during training to enhance generalization as well as the ability of inVAErt networks to deal with *practical* identifiability. In addition, we also leverage flow-based density estimation to perform inversion tasks with missing data, a typical feature of electronic health record (EHR) datasets.

Here, we briefly summarize our main contributions:

1. We perform data-driven model synthesis and generate an *enhanced digital twin* for a lumped‐parameter haemodynamic system (CVSim-6) using inVAErt networks.
2. We analyse in detail the stiffness of the system, showing that inVAErt networks can be used for model synthesis of stiff ODE systems.
3. We show the ability of an inVAErt network to characterize high-dimensional non-identifiable manifolds for complex systems of ODEs, and suggest a visualization technique that offers an improved assessment of the system identifiability.
4. We demonstrate the ability of inVAErt networks to handle practical non-identifiability and to work with real data from an EHR dataset with missing attributes.

The content of this article is organized as follows: In §1a, we briefly state the problem of interest and introduce the mathematical notations. The lumped-parameter haemodynamic model, the CVSim-6 system, is introduced in §2a, with its associated ODE stiffness analysis covered in §3. In §2b we discuss the architecture of our proposed neural networks, optimization tasks and training details. Two inference tasks are discussed in §3c,d. The first task focuses on using noiseless synthetic data to explore the structural identifiability of the CVSim-6 system, including the study of non-identifiable manifolds (§3c(ii)) and missing data analysis (§3c(iii)). The second task deals with real-world clinical measurements from an EHR dataset, and shows how the addition of training noise can effectively improve performance in inference tasks (§3d). For the interested reader, our dataset and code can be accessed at https://github.com/desResLab/InVAErt4Cardio.

### (a) Background and notation

The input-to-output (forward) map for a hypothetical cardiovascular model is denoted as f:V↦Y , mapping a vector of parameters $v\in V\subset {\mathbb{R}}^{\dim(v)}$ to a set of clinical targets $y\in Y\subset {\mathbb{R}}^{\dim(y)}$ , with V and Y representing the abstract input and output spaces, respectively. In this context, the operator f involves both solving a system of ODEs and post-processing quantities of clinical interest from the time-dependent solutions, e.g. extracting minimum, maximum (diastolic and systolic) or time-average values over the cardiac cycle, computing acceleration times, etc. The input v provides 0D, simplified characterization of the physiology through the hydrodynamic analogy [19], relating major viscous losses to Ohm resistance, vascular compliance to electric capacitance and blood flow inertia to inductance. Despite the simplicity of this approximation, varying v can span a broad spectrum of physiological conditions from healthy to disease [11,20].

Determining lumped‐parameter point estimates from observations (clinical data) is possible by solving the inverse problem v=h(y) , but, in practice, a number of challenges are associated with this task. First, the inverse or pseudo-inverse operator h:Y↦V is often ill-posed due to solution non-uniqueness in structurally non-identifiable models. Specifically, for a given output y , there may exist $M_{y}\subset V$ such that f(v)=y for all $v\in M_{y}$ . We call $M_{y}$ the *non-identifiable manifold*, embedded in V and associated with the observation y , where the complexity in determining this manifold depends on the nonlinearity of the forward map f , the properties of its gradient and the dimensionality mismatch between v and y [17]. The existence of $M_{y}$ is related to redundancy in the parameterization of the map f (or, in other words, the map f is not injective), as changes in one component of the input v can be entirely compensated by adjusting the remaining components, while leaving the output y unaffected [8,9,21].

Structural non-identifiability typically occurs in *partially observed* systems (or observed under the so-called *small data* regime) where dim⁡(y)<dim⁡(v) [8], and therefore, there is not enough information to uniquely determine the input parameters. Ill-posedness in inverse problems can be mitigated by additional observations, increasing the dimensionality of the output y . However, this may be impractical in clinical settings due to concerns related to risk or cost. By design, InVAErt networks [17] leverage input-dependent *latent variables* w∈W to compensate for this information gap (§2b), where, generally, a higher dimensional W increases the ability to capture complicated manifolds $M_{y}$ .

Second, ill-posedness of an inverse problem can also arise due to data scarcity, model misspecification or the presence of noise. All these factors lead to the violation of Hadamard’s conditions for well-posedness, that is, the existence of a unique solution that is stable [22–24]. In particular, real-life clinical data may not be adequately characterized by a simplified mathematical model or, formally, real measurements may be such that y∉Range(f) . This may be due to random noise originating from the finite precision of sensors and acquisition devices used in the clinic, or even human errors in reporting the data, which can push data outside the range of f significantly, increasing the complexity of inverse problems. Contrary to structural non-identifiability due to the inherent model parameterization, the above issues typically occur when dealing with real data, hence the name of *practical* non-identifiability [9].

Finally, input training data are generated from an assumed prior v∼p(v) which induces a distribution in the outputs f(v)=y∼p(y) . Sampling from and evaluating the output distribution p(y) are of critical importance when modelling physics-based systems. Therefore, inVAErt networks, besides learning the forward v↦y and inverse y↦v maps, are also equipped with a flow-based density estimator (§§3c(iii) and 3d).

## 2. Methods

### (a) The CVSim-6 haemodynamic model

**Figure 1.**
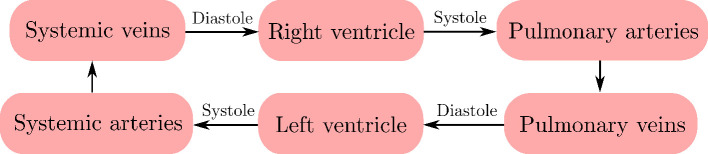
Schematic of compartments in the CVSim-6 model.

CVSim-6 is a six-compartment 0D model, originally proposed for teaching cardiovascular physiology using computers [6]. Additional studies related to the use of CVSim-6 for educational tool development, numerical simulation and parameter estimation can be found in [25–28]. It simulates the evolution of blood pressures, flows and volumes through the left and right ventricular chambers, systemic arteries, systemic veins, pulmonary arteries and pulmonary veins as shown in figure 1. The subscripts $(\cdot {)}_{l},(\cdot {)}_{r},(\cdot {)}_{a},(\cdot {)}_{v},(\cdot {)}_{pa},(\cdot {)}_{pv}$ refer to quantities specific to each compartment.

The input parameters $v\in {\mathbb{R}}^{23}$ are listed in table 1, with reference values listed from [6]. Each of the six compartments is characterized by a capacitance (compliance) C , a resistance R and an unstressed (zero-pressure filling) volume $V^{0}$ , except for the two ventricles, where prescribed systolic and diastolic capacitances are responsible for ventricular contraction and relaxation. Models for the atrial chamber are not included in CVSim-6. Consistent with the compartmental nature of the model and its formulation in terms of a closed-loop autonomous system of ODEs, we follow the convention where the outflow resistance of one compartment coincides with the inflow resistance of the subsequent compartment. Additional parameters include the heart rate *Hr *, transthoracic pressure $P_{th}$ (disregarding any circulatory–respiratory coupling) and the systolic fraction $r_{sys}$ of a heart cycle.

**Table 1.** Input parameters ( v ) for the CVSim-6 system.

num.	description	ref.	unit
1.	heart rate ( Hr )	72.00	(bpm)
2.	transthoracic pressure ( $P_{th}$ )	−4.00	(mm Hg)
3.	systolic ratio per heart cycle ( $r_{sys}$ )	0.33	—
4.	left ventricular diastolic capacitance ( $C_{l,dia}$ )	$7.50\times 10^{-3}$	(ml Barye^−1^)
5.	left ventricular systolic capacitance ( $C_{l,sys}$ )	$3.00\times 10^{-4}$	(ml Barye^−1^)
6.	arterial capacitance ( $C_{a}$ )	$1.20\times 10^{-3}$	(ml Barye^−1^)
7.	venous capacitance ( $C_{v}$ )	$7.50\times 10^{-2}$	(ml Barye^−1^)
8.	right ventricular diastolic capacitance ( $C_{r,dia}$ )	$1.50\times 10^{-2}$	(ml Barye^−1^)
9.	right ventricular systolic capacitance ( $C_{r,sys}$ )	$9.00\times 10^{-4}$	(ml Barye^−1^)
10.	pulmonary arterial capacitance ( $C_{pa}$ )	$3.23\times 10^{-3}$	(ml Barye^−1^)
11.	pulmonary venous capacitance ( $C_{pv}$ )	$6.30\times 10^{-3}$	(ml Barye^−1^)
12.	left ventricular input resistance ( $R_{l,in}$ )	13.33	(Barye s ml^−1^)
13.	left ventricular output resistance ( $R_{l,out}$ )	8.00	(Barye s ml^−1^)
14.	arterial resistance ( $R_{a}$ )	1333.22	(Barye s ml^−1^)
15.	right ventricular input resistance ( $R_{r,in}$ )	66.66	(Barye s ml^−1^)
16.	right ventricular output resistance ( $R_{r,out}$ )	4.00	(Barye s ml^−1^)
17.	pulmonary venous resistance ( $R_{pv}$ )	106.66	(Barye s ml^−1^)
18.	unstressed left ventricular volume ( $V_{l}^{0}$ )	15.00	(ml)
19.	unstressed arterial volume ( $V_{a}^{0}$ )	715.00	(ml)
20.	unstressed venous volume ( $V_{v}^{0}$ )	2500.00	(ml)
21.	unstressed right ventricular volume ( $V_{r}^{0}$ )	15.00	(ml)
22.	unstressed pulmonary arterial volume ( $V_{pa}^{0}$ )	90.00	(ml)
23.	unstressed pulmonary venous volume ( $V_{pv}^{0}$ )	490.00	(ml)

The CVSim-6 system is governed by the six ODEs in equation (2.1), one per compartment, with six pressures as state variables, i.e. $\left[P_{l},P_{a},P_{v},P_{r},P_{pa},P_{pv}\right]$ the evolutions of which are governed by a combination of Kirchhoff’s law, two-element Windkessel models and four ideal (no regurgitation) unidirectional heart valves, as detailed in [10]. Generalized Ohm equations between pressures and flows are reported in equation (2.2), where $I_{\left(\cdot \right)}$ denotes the indicator function, and the time-dependent ventricular compliances $C_{l}(t)$ , $C_{r}(t)$ are defined in appendix A.

Solving the above ODE system also requires proper specification of initial conditions for the pressure in each compartment. This is accomplished through the solution of a linear system of equations that ensures mass conservation at t=0 . For additional information, interested readers may refer to [6,26], or equation (A 1) in appendix A.


(2.1) $$\left\{ \begin{array}{rl} {\dot{P}}_{l}(t) & =\frac{Q_{l,in}(t)-Q_{l,out}(t)-(P_{l}(t)-P_{th})\dot{C_{l}}(t)}{C_{l}(t)},\\ {\dot{P}}_{a}(t) & =\frac{Q_{l,out}(t)-Q_{a}(t)}{C_{a}},\\ {\dot{P}}_{v}(t) & =\frac{Q_{a}(t)-Q_{r,in}(t)}{C_{v}},\\ {\dot{P}}_{r}(t) & =\frac{Q_{r,in}(t)-Q_{r,out}(t)-(P_{r}(t)-P_{th}){\dot{C}}_{r}(t)}{C_{r}(t)},\\ {\dot{P}}_{pa}(t) & =\frac{Q_{r,out}(t)-Q_{pv}(t)}{C_{pa}},\\ {\dot{P}}_{pv}(t) & =\frac{Q_{pv}(t)-Q_{l,in}(t)}{C_{pv}}. \end{array}\right.$$

(2.2) $$\left\{ \begin{array}{rl} Q_{l,in}(t) & =\frac{P_{pv}(t)-P_{l}(t)}{R_{l,in}}I_{P_{pv}(t)>P_{l}(t)},\\ Q_{l,out}(t) & =\frac{P_{l}(t)-P_{a}(t)}{R_{l,out}}I_{P_{l}(t)>P_{a}(t)},\\ Q_{a}(t) & =\frac{P_{a}(t)-P_{v}(t)}{R_{a}},\\ Q_{r,in}(t) & =\frac{P_{v}(t)-P_{r}(t)}{R_{r,in}}I_{P_{v}(t)>P_{r}(t)},\\ Q_{r,out}(t) & =\frac{P_{r}(t)-P_{pa}(t)}{R_{r,out}}I_{P_{r}(t)>P_{pa}(t)},\\ Q_{pv}(t) & =\frac{P_{pa}(t)-P_{pv}(t)}{R_{pv}}. \end{array}\right.$$

### (b) InVAErt networks

**Figure 2.**
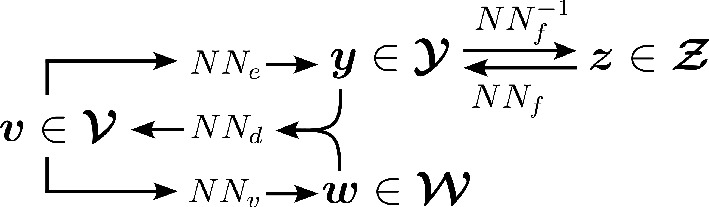
Schematic of all components of an inVAErt network and their interactions.

InVAErt networks [17] provide a comprehensive, data-driven methodology for physics-based digital twins (diagram in figure 2). They consist of (i) an emulator $NN_{e}:V\mapsto Y$ as a surrogate for the input-to-output map f , (ii) a L -layer flow-based density estimator $NN_{f}:Z\mapsto Y$ mapping a tractable base distribution $\pi_{0}(z)$ , usually a standard Gaussian, to the output target distribution p(y) , (iii) a variational encoder $NN_{v}:V\mapsto W$ generating an input-dependent latent space responsible for the lack of bijectivity between inputs and outputs. Finally, (iv) a decoder $NN_{d}:Y\times W\mapsto V$ approximates the *inverse* map from combined outputs and latent variables to model inputs.

Given a dataset $D=\{(v_{j},y_{j}){\}}_{j=1}^{N}$ , optimal weights and biases for each inVAErt network component are obtained by minimizing


(2.3) $$\left\{ \begin{array}{ll} \phi_{e}^{opt} & =\underset{\phi_{e}}{\mathrm{argmin}}\sum_{j=1}^{N}{\left\|y_{j}-NN_{e}\left(v_{j};\phi_{e}\right)\right\|}_{2}^{2},\\ \phi_{f}^{opt} & =\underset{\phi_{f}}{\mathrm{argmax}}\sum_{j=1}^{N}\left(\log\pi_{0}\left(z_{j}^{\left(0\right)}\right)-\sum_{l=1}^{L}\log\left|\det\left(\frac{dz_{j}^{\left(l\right)}}{dz_{j}^{\left(l-1\right)}}\right)\right|\right),\\ \phi_{v}^{opt},\phi_{d}^{opt} & =\underset{\phi_{v},\phi_{d}}{\mathrm{argmin}}[\beta_{d}\sum_{j=1}^{N}{\left\|v_{j}-NN_{d}\left(y_{j},w_{j};\phi_{d}\right)\right\|}_{2}^{2}+\frac{\beta_{v}}{2}\sum_{j=1}^{N}\sum_{k=1}^{\dim(w)}\left(\mu_{jk}^{2}+\sigma_{jk}^{2}-\log\left(\sigma_{jk}^{2}\right)-1\right)\\ & +\beta_{r}\sum_{j=1}^{N}{\left\|y_{j}-NN_{e}\left(NN_{d}\left(y_{j},w_{j};\phi_{d}\right);\phi_{e}^{opt}\right)\right\|}_{2}^{2}], \end{array}\right.$$

which combines a mean squared error (MSE) loss for the emulator $NN_{e}$ , a maximum likelihood estimation loss for the normalizing flow density estimator $NN_{f}$ and joint MSE and Kullback–Leibler (KL)-divergence losses for the variational encoder $NN_{v}$ and the decoder $NN_{d}$ .

We utilize a real-valued non-volume preserving transformation (Real-NVP) discrete normalizing flows architecture [29], which consists of L bijections defined as


$$NN_{f}(z^{\left(0\right)};\phi_{f})=(g_{L}\circ \cdots \circ g_{2}\circ g_{1})(z^{\left(0\right)}),\;z^{\left(0\right)}∼\pi_{0}(z)\;,$$

where $z^{\left(l\right)}=g_{l}(z^{\left(l-1\right)}),z^{\left(l-1\right)}=g_{l}^{-1}(z^{\left(l\right)}),l=1,\ldots ,L$ and $z^{\left(L\right)}\approx y∼p(y)$ . Additional details on approaches for discrete and continuous normalizing flows can be found in [30,31].

The losses associated with the variational encoder and dense decoder are penalized with constants $\beta_{v}$ and $\beta_{d}$ , respectively. Generation of input-dependent latent variables w∼p(w|v) follows the classical VAE framework [32], i.e.


(2.4) $$NN_{v}(v;\phi_{v})=[\mu ,\log\sigma^{2}{]}^{T},\;w=\mu +\epsilon ⊙\sigma ,\;\epsilon ∼N(0,I)\;,$$

and the KL-divergence regularizes the latent space W by reducing the statistical distance between the posterior distribution p(w|v) and a standard normal prior p(w) . Finally, the output reconstruction loss (sometimes referred to as the *re-evaluation or re-constraining* loss [17]) uses the previously trained emulator, i.e. $NN_{e}(\cdot ,\phi_{e}^{\text{opt}})$ , to make sure decoded parameters lead to model outputs that are close to the observations used as inputs to the decoder. This loss is penalized by the constant $\beta_{r}$ in equation (2.3).

#### (i) Noise injection as training data augmentation

To tackle challenging inference tasks involving practical non-identifiability, we add artificial Gaussian noise to the labels y during the training for $NN_{f}$ , $NN_{v}$ and $NN_{d}$ . Incorporating training noise as a form of data augmentation has been shown to improve model generalization and reduce overfitting [33,34]. This is helpful in inference tasks when dealing with out-of-distribution data, measurement noise and model misspecification (§3d)*.*

Note that the forward map f is assumed to be deterministic in this study, hence no noise injection is performed when training the neural emulator $NN_{e}$ . However, when training all other inVAErt network components, we consider the output labels corrupted by zero-mean, uncorrelated and heteroskedastic Gaussian noise, i.e.


(2.5) $$\begin{array}{lcr} & y^{\prime }=y+\eta ,\;\eta ∼N(0,\text{diag}(s^{2})),\;s^{2}=[s_{1}^{2},\cdots \;,s_{\dim(y)}^{2}{]}^{T}\;, & \end{array}$$

with known standard deviation values for each output component. The random noise is independent of both the input and output data, with a new realization generated at each epoch. In summary, $y^{\prime }$ replaces y in all terms in equation (2.3), except for the MSE emulator loss.

## 3. Numerical experiments

In this section, we provide a number of new results for inVAErt networks in the context of computational physiology. We first characterize the stiffness of the differential system used throughout this section, showing that lumped‐parameter models give rise to stiff systems of ODEs. We also highlight the most important mechanisms that are responsible for this stiffness. We then use synthetically generated data from a baseline set of parameters (the so-called *default* parameter set) to show the ability of inVAErt networks to identify entire manifolds of non-identifiable parameters and provide ways to graphically characterize such manifolds. Finally, we show how to select the parameter prior, the amount of noise and how to deal with missing data, demonstrating the use of inVAErt networks for parametric inversion in the presence of practical non-identifiability.

### (a) Stiffness analysis

Like other pulsatile cardiovascular models driven by periodic forcing [7], the CVSim-6 system exhibits stiffness and may suffer from numerical instability without a careful selection of the integration time step. To formally study this phenomenon, we first write equation (2.1) in matrix form:


(3.1) $$\begin{array}{lcr} & \dot{P}(t)=A(t)\;P(t)+b(t)\;, & \end{array}$$

where $P(t)=[P_{l}(t),P_{a}(t),P_{v}(t),P_{r}(t),P_{pa}(t),P_{pv}(t){]}^{T}$ represents the state vector, A(t) denotes the time-dependent coefficient matrix and b(t) is a time-varying forcing vector which quantifies the contribution of the intrathoracic pressure [6].

Rather than following a rigorous definition, stiffness in ODE systems is often identified by its effects on numerical solutions [35–37]. For the current system, one way to analyse stiffness is through the eigen-decomposition of the matrix A(t) , i.e. $A(t)=Q_{t}\Lambda_{t}Q_{t}^{-1}$ . Here, $\Lambda_{t}\in {\mathbb{C}}^{6\times 6}$ is a diagonal matrix with entries equal to the eigenvalues $\lambda_{1}(t),\ldots ,\lambda_{6}(t)$ , with $|\text{Re}(\lambda_{1}(t))|\geq \cdots \geq |\text{Re}(\lambda_{6}(t))|$ , and $Q_{t}\in {\mathbb{C}}^{6\times 6}$ is the matrix of eigenvectors, i.e. $Q_{t}=[Q_{1}(t),\ldots ,Q_{6}(t)]$ . As A(t) depends on the state vector P(t) via the indicator functions of equation (2.2), A(t) is approximated using a fifth-order implicit Runge–Kutta method (Radau) with adaptive step [38] as discussed in [6]. The simulation runs for 12 cardiac cycles, i.e. $12\cdot T_{tot}=12\cdot 60/Hr$ , utilizing the *default* set of input parameters listed in table 1.

**Figure 3.**
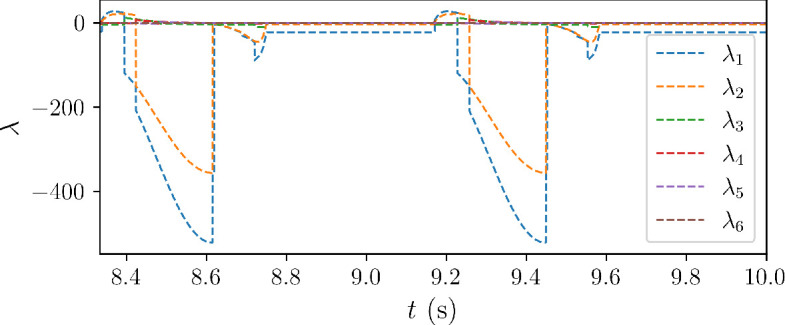
Time history of the eigenvalues of A(t) plotted over two heart cycles.

All six eigenvalues of A(t) are real, with time histories over the last two heart cycles shown in figure 3. The first two modes are associated with the largest magnitudes occurring at the same time during each heart cycle.

At any time t , the *stiffness ratio* SR (t) is defined as


(3.2) $$\text{SR}(t)=\frac{\max|\text{Re}(\Lambda_{t})|}{\min|\text{Re}(\Lambda_{t})|}\;,$$

and can inform on the *degree* of stiffness of a given system. Specifically, a large SR implies a significant difference in the rate of change between solution components, so that a smaller time step is required to capture both fast- and slow-varying dynamics [35–37,39]. In addition, it is possible for $\min|\text{Re}(\Lambda_{t})|$ to be zero for some t (figure 3), indicating a solution component remaining constant (e.g. due to a time-varying capacitance *driving* the autonomous system response). In such a case, the denominator in equation (3.2) is replaced by the non-zero eigenvalue with the smallest magnitude, using a tolerance *tol* ( $tol=1\times 10^{-14}$ is used here, remark 1).

**Figure 4.**
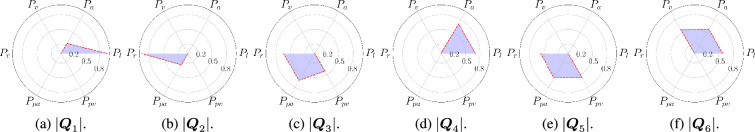
Radar plots of absolute eigenvector components for maximum SR (t) at t∼9.444 (s). Associated eigenvalues: $\lambda_{1}=-520.95$ , $\lambda_{2}=-355.93$ , $\lambda_{3}=-3.75$ , $\lambda_{4}=-0.51$ , $\lambda_{5}=6.25\cdot 10^{-4}$ , $\lambda_{6}=2.91\times 10^{-5}$ .

**Figure 5.**
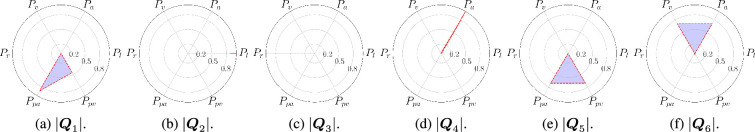
Radar plots of absolute eigenvector components for minimum SR (t) at t∼8.618 (s). Associated eigenvalues: $\lambda_{1}=-4.40$ , $\lambda_{2}=-1.81$ , $\lambda_{3}=-1.77$ , $\lambda_{4}=-0.64$ , $\lambda_{5}=2.22\cdot 10^{-16}$ , $\lambda_{6}=3.47\times 10^{-18}$ .

In figures 4 and 5, we also show the absolute components of each eigenvector and report the corresponding eigenvalue for times at which the stiffness ratio SR (t) reaches its minimum and maximum value, respectively, within the last two heart cycles.

The maximum stiffness is observed when the magnitudes of $\lambda_{1}$ and $\lambda_{2}$ are maximized (figures 3 and 4), with intrinsic timescale constants equal to [39]


$$\tau_{1}=1/|\lambda_{1}|=0.0019\;(\text{s}),\;\tau_{2}=1/|\lambda_{2}|=0.0028\;(\text{s}).$$

**Figure 6.**
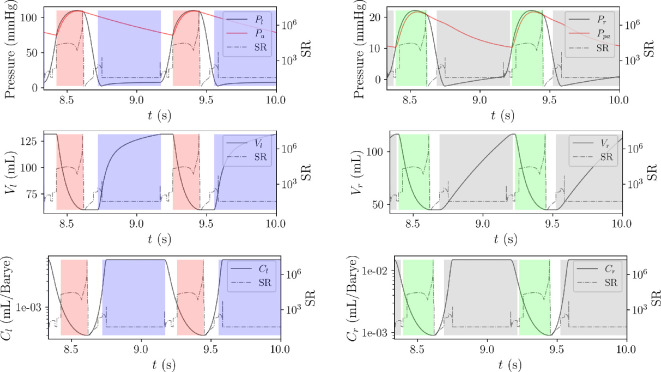
Variation of stiffness ratio SR (t) over two heart cycles. The CVSim-6 dynamics is also shown in terms of ventricular volumes ( $V_{l}$ , $V_{r}$ ), ventricular pressures ( $P_{l}$ , $P_{r}$ ), arterial pressure ( $P_{a}$ ), pulmonary arterial pressure ( $P_{pa}$ ) and time-varying ventricular capacitance ( $C_{l},C_{r}$ ). The opening and closing intervals for the mitral (blue), aortic (red), tricuspid (grey) and pulmonary (green) valves are depicted using shaded colours.

These values agree well with resistor–capacitor (RC) constants computed at the outflow of the left and right ventricles, respectively (Table 4.2 of [6]), defined as $RC_{j,k}=R_{j,k}C_{j}C_{k}/(C_{j}+C_{k})$ for neighbouring compartments j and k [6]. Analogous to RC electric circuits, the RC constant τ serves as a characteristic timescale, quantifying the speed at which a haemodynamic compartment responds to excitations or settles into steady-state conditions once external forcing is removed. A small τ indicates a rapidly evolving dynamic response, which needs to be resolved through an appropriate time‐step size. In addition, the largest difference between solution components occurs due to ventricular–arterial coupling (see $|Q_{5}|$ and $|Q_{6}|$ in figure 4), and is observed just before the closure of the aortic and pulmonary valve in late systole (figure 6).

Figure 6 also shows how the stiffness ratio SR (t) varies with respect to CVSim-6 system dynamics, specifically right and left ventricular pressure, volume, time-varying compliance and valve opening times; SR increases significantly during systole, peaking just before the aortic and pulmonary valves close. It then drops sharply to its minimum at the onset of isovolumetric relaxation, and remains low throughout the rest of diastole.

**Figure 7.**
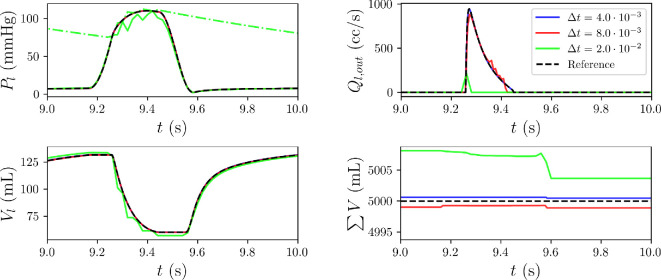
Comparison between numerical approximations for left ventricular pressure ( $P_{l}$ ), outflow ( $Q_{l,out}$ ), volume ( $V_{l}$ ) and total stressed volume ( ∑V ). We compare an explicit fourth-order Runge–Kutta method with three different time‐step sizes equal to $\Delta t=4\times 10^{-3}$ (s), $8\times 10^{-3}$ (s) and $2\times 10^{-2}$ (s). The reference approach is an implicit fifth-order Runge–Kutta method (Radau [38]) with adaptive step size. The systemic arterial pressure solution $P_{a}$ when $\Delta t=2\times 10^{-2}$ (s) is also super-imposed in the top-left figure as the green dash-dotted line.

Finally, figure 7 shows how the use of an inappropriate time‐step size can trigger numerical instability and a non-physiological response. Specifically, when $\Delta t=2\times 10^{-2}$ (s), an inaccurate approximation of the left ventricular pressure $P_{l}$ makes it lower than the systemic arterial pressure $P_{a}$ during systole (where a high stiffness ratio is observed), nearly causing the aortic valve to close. The figure also shows that an appropriate time step and numerical algorithms are needed to ensure the conservation of total stressed volume ∑V (see also appendices A and E for more information).

In conclusion, stiffness in ODE systems can pose challenges in both data-driven forward emulation and inverse modelling, since simulated data of poor quality may result from situations where the time‐step size or the numerical algorithm are not carefully selected, as shown in figure 7. Also, an inadequate numerical solution can inject practical non-identifiability into the inference problem, even if no measurement noise is present in the data acquisition process.

**Remark 1.** *The stiffness of an ODE system can be overestimated by SR. For example, if* $|\lambda_{\max}|=0.1$ *and* $|\lambda_{\min}|=1\times 10^{-11}$ *, SR* $=1\times 10^{10}$ *while the fastest timescale* $\tau =1/|\lambda_{\max}|$ *remains fairly large. In addition, computation of small eigenvalues is affected by round-off error, numerical solver accuracy, etc. An alternative criterion to measure stiffness of an ODE system is through the Lipschitz constant [* 35,36 *], which is bounded below by the spectral radius, i.e. the magnitude of the largest eigenvalue for the right-hand side Jacobian. Therefore, as discussed above, we set a certain tolerance for the smallest eigenvalue to compute the stiffness ratio, but also specifically pay attention to the largest eigenvalue alone when characterizing stiffness.*

**Remark 2.** *As a result of assuming perfect synchronization between the left and right ventricular time-varying capacitance in* equation (A 2) *, the duration of systole and diastole are expected to be identical for the two ventricles. However, due to differences in ventricular pressure and volume, and numerical accuracy, a small delay can occur between heart cycles as seen from the left and right ventricles (e.g.* *figure 6* *). This delay may depend on the specific input parameter combination, but in general is very limited. Therefore, for simplicity, we do not take it into consideration when calculating the right ventricular end-diastolic pressure* $P_{r,edp}$ *.*

### (b) Output selection

The selection of output attributes $y\in {\mathbb{R}}^{16}$ for the CVSim-6 system (table 2) is based on the EHR dataset [11] to be studied in §3d. This dataset includes patient-specific measurements of heart rate, systolic and diastolic blood pressures, mean venous pressure, left ventricular volumes, cardiac output and vascular resistance. Corresponding model outputs are computed from the CVSim-6 solutions over the final three of 12 simulated heart cycles (we refer to these three cycles as $T_{p}$ with size $n_{p}$ ) due to a fully converged periodic response.

**Table 2.** CVSim-6 system outputs ( y ) and corresponding uncertainty.

num.	description	calculation	unit	std.
1.	heart rate ( Hr )	copied from input	(bpm)	3.0
2.	systolic blood pressure ( $P_{a,sys}$ )	$\max_{t\in T_{p}}P_{a}(t)$	(mm Hg)	1.5
3.	diastolic blood pressure ( $P_{a,dia}$ )	$\min_{t\in T_{p}}P_{a}(t)$	(mm Hg)	1.5
4.	right ventricular systolic pressure ( $P_{r,sys}$ )	$\max_{t\in T_{p}}P_{r}(t)$	(mm Hg)	1.0
5.	right ventricular diastolic pressure ( $P_{r,dia}$ )	$\min_{t\in T_{p}}P_{r}(t)$	(mm Hg)	1.0
6.	pulmonary arterial systolic pressure ( $P_{pa,sys}$ )	$\max_{t\in T_{p}}P_{pa}(t)$	(mm Hg)	1.0
7.	pulmonary arterial diastolic pressure ( $P_{pa,dia}$ )	$\min_{t\in T_{p}}P_{pa}(t)$	(mm Hg)	1.0
8.	right ventricular end-diastolic pressure ( $P_{r,edp}$ )	$P_{r}(T_{p}[n_{p}])$	(mm Hg)	1.0
9.	pulmonary wedge pressure ( $P_{w}$ )	$\frac{1}{|T_{p}|}\int_{T_{p}}P_{pv}(t)\;dt$	(mm Hg)	1.0
10.	central venous pressure ( $P_{cvp}$ )	$\frac{1}{|T_{p}|}\int_{T_{p}}P_{v}(t)\;dt$	(mm Hg)	0.5
11.	systolic left ventricular volume ( $V_{l,sys}$ )	$\min_{t\in T_{p}}V_{l}(t)$	(mL)	10.0
12.	diastolic left ventricular volume ( $V_{l,dia}$ )	$\max_{t\in T_{p}}V_{l}(t)$	(mL)	20.0
13.	left ventricular ejection fraction (LVEF)	$(V_{l,dia}-V_{l,sys})/V_{l,dia}$	−	0.02
14.	cardiac output (CO)	$\frac{1}{|T_{p}|}\int_{T_{p}}Q_{a}(t)\;dt$	(L/min)	0.2
15.	systemic vascular resistance (SVR)	$(\frac{1}{|T_{p}|}\int_{T_{p}}P_{a}(t)\;dt-P_{cvp})/\text{CO}$	(dyn ⋅ s cm^−5^)	50.0
16.	pulmonary vascular resistance (PVR)	$(\frac{1}{|T_{p}|}\int_{T_{p}}P_{pa}(t)\;dt-P_{w})/\text{CO}$	(dyn ⋅ s cm^−5^ )	5.0

Note that the pulmonary wedge pressure $P_{w}$ and central venous pressure $P_{cvp}$ are indirect measures for the left and right atrial pressures [40], respectively, despite the absence of atrial compartments in CVSim-6. Furthermore, the stroke volume (SV) is calculated as the difference between diastolic and systolic volumes in the left ventricle, i.e. $\text{SV}=V_{l,dia}-V_{l,sys}$ and from which, the cardiac output can (CO) be alternatively estimated as CO=SV×Hr (e.g. [40]). Finally, to account for measurement noise, we use standard deviations for each output component as listed in table 2, based on values suggested in the literature [11,41–43].

### (c) Structural non-identifiability analysis with synthetic data

We first investigate structural non-identifiability in CVSim-6 using noiseless synthetic data. A set of N = 54 000 input–output pairs is generated by numerically solving the CVSim-6 system, and then subdivided into 37 500 training data, 12 500 online testing data and 4000 offline validation data. Input parameters are randomly selected from a uniform prior, centred around the default parameter combination (table 1). Specifically, the compliance parameters are allowed to vary by up to ±50% , and other input parameters can fluctuate up to ±30% , relative to their default values. For brevity, we summarize the results of the forward emulation and output density estimation in appendix C.1 and focus on the inverse problem in this section. The choice of hyperparameters and other training details can be found in appendix B.1.

We first validate the ability of an inVAErt network to solve amortized inverse problems by checking if a given output y can be reconstructed via the decoded parameters $\hat{v}$ . To do so, we extract all 4000 labels from the validation dataset and sample an equal number of latent variables from the standard normal, i.e. w∼N(0,I) . Then, concatenated outputs and latent space samples $[y,w{]}^{T}$ are fed to the trained decoder $NN_{d}$ , resulting in 4000 inverse predictions $\hat{v}$ . Finally, we evaluate these parameters through the exact CVSim-6 simulator, i.e. $\hat{y}=f(\hat{v})$ , and record the absolute reconstruction difference $|y-\hat{y}|$ of each output component in table 3. Errors are very limited on average. In particular, we observe a maximum absolute error in the systolic arterial pressure equal to 3.34 mm Hg, due to either an output y or a latent variable w belonging to the tail of the respective distribution.

**Table 3.** Statistics of the absolute reconstruction error across the validation dataset of size 4000.

	Hr (bpm)	$P_{a,sys}$ (mm Hg)	$P_{a,dia}$ (mm Hg)	$P_{r,sys}$ (mm Hg)	$P_{r,dia}$ (mm Hg)
average	4.62 × 10^−2^	3.37 × 10^−1^	2.50 × 10^−1^	8.16 × 10^−2^	1.12 × 10^−2^
max	4.06 × 10^−1^	3.34 × 10^+0^	2.42 × 10^+0^	1.60 × 10^+0^	1.41 × 10^−1^
SD	4.36 × 10^−2^	3.32 × 10^−1^	2.36 × 10^−1^	8.27 × 10^−2^	1.20 × 10^−2^

	$P_{pa,sys}$ (mm Hg)	$P_{pa,dia}$ (mm Hg)	$P_{r,edp}$ (mm Hg)	$P_{w}$ (mm Hg)	$P_{cvp}$ (mm Hg)
average	8.06 × 10^−2^	5.12 × 10^−2^	1.82 × 10^−2^	4.89 × 10^−2^	2.72 × 10^−2^
max	1.58 × 10^+0^	1.08 × 10^+0^	2.78 × 10^−1^	1.12 × 10^+0^	3.26 × 10^−1^
SD	8.16 × 10^−2^	5.48 × 10^−2^	1.93 × 10^−2^	5.20 × 10^−2^	2.71 × 10^−2^

	$V_{l,sys}$ (ml)	$V_{l,dia}$ (ml)	LVEF (−)	CO (l min^−1^)	SVR (dyn s cm^−5^)	PVR (dyn s cm^−5^)
average	1.49 × 10^−1^	3.29 × 10^−1^	5.04 × 10^−4^	1.41 × 10^−2^	1.02 × 10^+0^	8.79 × 10^−2^
max	1.79 × 10^+0^	4.67 × 10^+0^	6.95 × 10^−3^	2.22 × 10^−1^	1.20 × 10^+0^	6.54 × 10^−1^
SD	1.57 × 10^−1^	3.41 × 10^−1^	5.09 × 10^−4^	1.44 × 10^−2^	9.32 × 10^−1^	8.07 × 10^−2^

#### (i) Going beyond point estimates

In the previous section, each output y was associated with a single latent variable realization w , thereby generating *a single* inverse problem solution. However, structurally non-identifiable systems are characterized by the possibility that infinitely many inputs $v\in M_{y}\subset V$ may map to a common output y , i.e. f(v)=y for any $v\in M_{y}$ . For an inVAErt network, discovering multiple solutions from the non-identifiable manifold $M_{y}$ is as easy as drawing more latent samples from W , and performing one decoder evaluation for each of these samples. To test this, we first select a representative output from the trained density estimator $NN_{f}$ , denoted as $y^{*}$ , with its components listed in table 4.

**Table 4.** Selected CVSim-6 output $y^{*}$ sampled from $NN_{f}$ .

Hr (bpm)	$P_{a,sys}$ (mm Hg)	$P_{a,dia}$ (mm Hg)	$P_{r,sys}$ (mm Hg)	$P_{r,dia}$ (mm Hg)	$P_{pa,sys}$ (mm Hg)
72.91	142.78	111.55	22.78	−1.51	22.37

$P_{pa,dia}$ (mm Hg)	$P_{r,edp}$ (mm Hg)	$P_{w}$ (mm Hg)	$P_{cvp}$ (mm Hg)	$V_{l,sys}$ (ml)
13.13	1.61	12.56	7.27	76.81

$V_{l,dia}$ (mL)	LVEF (−)	CO (l min^−1^)	SVR (dyn s cm^−5^)	PVR (dyn s cm^−5^)
153.61	0.50	5.60	1725.48	74.53

**Figure 8.**
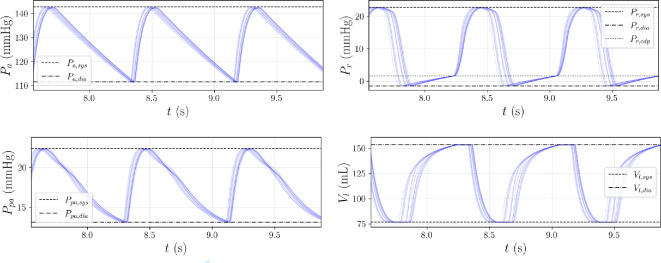
Trajectories of systemic arterial pressure $P_{a}$ , right ventricular pressure $P_{r}$ , pulmonary arterial pressure $P_{pa}$ and left ventricular volume $V_{l}$ corresponding to different latent space samples, computed from the observation $y^{*}$ (table 4). To better visualize each reconstruction, we plot only 20/100 pressure and volume time traces, together with systolic/diastolic targets in $y^{*}$ using horizontal lines.

For this output, we draw 100 samples w from the latent space and process each $[y^{*},w{]}^{T}$ through $NN_{d}$ , generating 100 predictions of $\hat{v}$ . To confirm these predictions are indeed associated with the non-identifiable manifold $M_{y^{*}}$ , we use them to reconstruct $y^{*}$ via the exact numerical simulator f , achieving a maximum relative error of approximately 0.139%. When compared with the components in $y^{*}$ as presented in figure 8, the pressure/volume trajectories computed through $\hat{v}$ , are able to accurately reproduce both systolic and diastolic targets.

#### (ii) Analysis of non-identifiable manifolds

**Figure 9.**
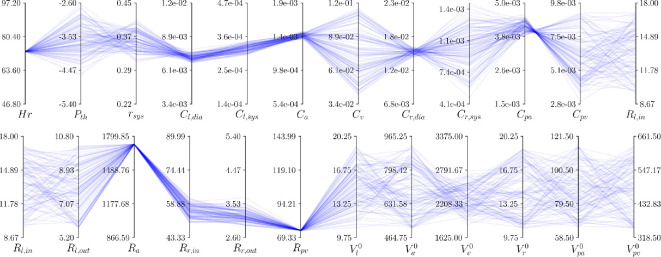
Parallel coordinate plot of the non-identifiable manifold $M_{y^{*}}$ , obtained by inverting the fixed observation $y^{*}$ (table 4), together with 100 latent variable realizations w drawn from a standard normal. Plot limits for each parameter are ±5% larger than the bounds defined in §3c, with units omitted for brevity (table 1). The parameter $R_{l,in}$ is repeated in both rows to include all connections.

A parallel coordinate plot of the manifold $M_{y^{*}}$ is presented in figure 9. First, a significant variability can be observed in some of the input components, confirming that the CVSim-6 system is structurally non-identifiable. From the picture, parameters such as $C_{v},C_{r,sys},C_{pv}$ , $R_{l,in},R_{l,out}$ and most of the unstressed volumes appear to be mostly responsible for the lack of identifiability, as their predicted values cover almost entirely their prior ranges. For unstressed volumes, this relates to the conservation of the total blood volume, which is enforced to determine the initial conditions for the CVSim-6 system equation (A 1). However, input parameters such as $R_{a}$ , $R_{pv}$ show very limited variations. This suggests the non-identifiable manifold $M_{y^{*}}$ embedded in the 23-dimensional input space V may be characterized by a smaller intrinsic dimension and, in turn, that some of the parameters are actually identifiable. For an additional discussion on parameter variability, please refer to appendix D.

**Figure 10.**
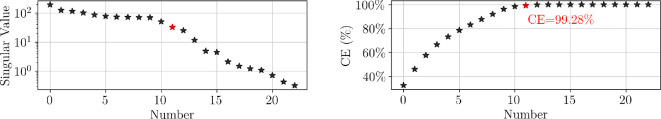
Singular value spectrum and cumulative energy distribution of 5000 input parameters belonging to $M_{y^{*}}$ .

To discover the lower dimensional structure embedded in $M_{y^{*}}$ , we compute the singular values of the data matrix sampled from $M_{y^{*}}$ , and the associated *cumulative energy* (CE) [44,45] (figure 10). CE measures how much data variance is restored by keeping the first n principal components. It is the ratio of the sum of the first n squared singular values, to the sum of the squares of all singular values. As illustrated in figure 10, 12 modes are sufficient to reconstruct 99.28% of the total *energy* [45].

#### (iii) Missing data analysis

Consider now the situation where the components $\left\{P_{a,dia},P_{r,dia},P_{pa,dia},P_{w},V_{l,sys},\text{SVR},\text{PVR}\right\}$ of $y^{*}$ are missing, e.g. unobserved. We wonder whether one can infer these missing elements through the pre-trained density estimator $NN_{f}$ , and then solve the inverse problem using the decoder network $NN_{d}$ . We answer this question using Algorithm 1. The ranking mechanism in Algorithm 1 promotes certain combinations of components that are most frequently observed in the solution of the CVSim-6 system, providing an approach for physics-based multiple imputation (e.g. [46]).

**Figure:**
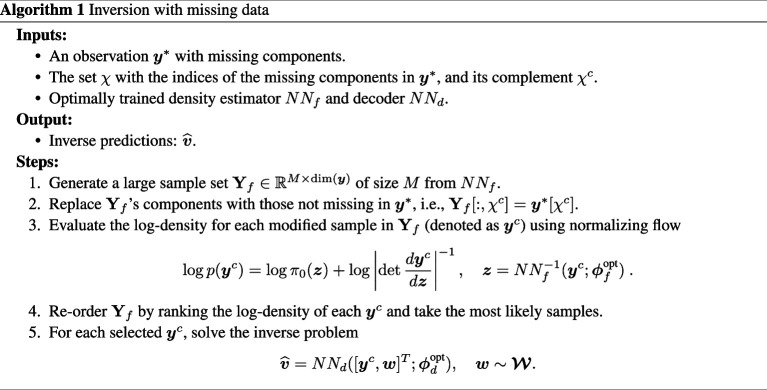


**Figure 11.**
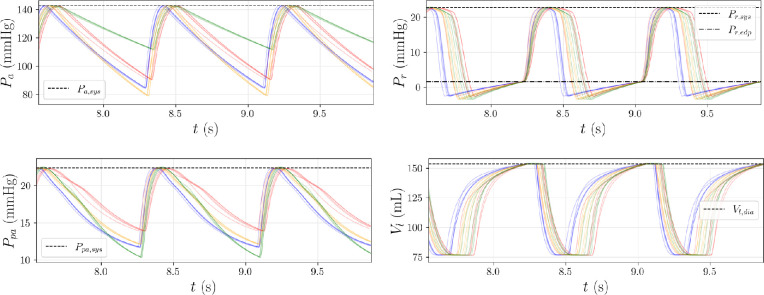
Trajectories of systemic arterial pressure $P_{a}$ , right ventricular pressure $P_{r}$ , pulmonary arterial pressure $P_{pa}$ and left ventricular volume $V_{l}$ obtained from $\hat{v}$ under missing data. Four potential outputs are extracted based on the density ranking, with five latent samples of w utilized for the inverse problem in each group. Different colours are applied to each group, and the target (non-missing) systolic/diastolic components of $y^{*}$ are plotted using horizontal lines.

To show the accuracy of this approach, we remove the previously mentioned components (assumed missing) from $y^{*}$ , and extract the top four most probable outputs using Algorithm 1. Next, for each output, we solve the inverse problem by sampling five latent variables w from the standard normal, resulting in four groups of inverse predictions of $\hat{v}$ . We then evaluate these parameters with the exact CVSim-6 simulator f , and plot the resulting pressure/volume curves in figure 11, using a different colour for each group.

First, the systolic and diastolic extrema for the trajectories in figure 11 align closely with the corresponding targets (non-missing) in $y^{*}$ , indicating a high reconstruction accuracy (maximum relative reconstruction error based on the 4×5=20 predictions is approximately 0.408% ). Second, the missing diastolic components for the systemic and pulmonary arterial pressures in $y^{*}$ are free to change between values with high likelihood. In contrast, the right ventricular diastolic pressures show only minor variations, and the left ventricular systolic volumes remain relatively consistent across each group. This behaviour is specific for the selected $y^{*}$ and may change with other observations and different missing components.

### (d) Inverse problem with real clinical measurements

We now consider data from a real EHR dataset provided through Google’s ATAP research project [11,25], containing clinical measurements from 84 fully anonymized adult patients. Each patient has up to 26 features, but none of the patients has data for all the features. While the missing components vary among the cohort, attributes such as heart rate or systolic and diastolic blood pressures are available for almost all patients, since they can be easily measured. As mentioned in §3b, 16 out of these 26 clinical targets are selected as outputs for the CVSim-6 system in this article.

Unlike studies with synthetic data in the previous sections, inferring input parameters through these real patient-specific data is a challenging task, mainly due to (i) potential measurement noise, (ii) missing data in each measurement, and (iii) simplifying assumptions and model misspecification in CVSim-6 with respect to the real cardiovascular physiology. In other words, an EHR measurement may not belong to the CVSim-6 model range (also known as out-of-distribution data). As a result, the complexity of the inversion tasks may increase significantly due to combined structural and practical non-identifiability.

To handle these challenges, we made three main adjustments. First, we expanded the bounds for the prior used to select training inputs (compared to §3c) such that the corresponding CVSim-6 outputs would cover most of the clinical measurements in the EHR dataset. Specifically, we allowed the heart rate to vary from −20 to +60% , and each compliance and resistance to vary from −80 to +60% , and the remaining parameters to vary by ±30% , with respect to their default values listed in table 1. Second, we added noise during network training to enhance generalization. The noise for each output component is modelled as a zero-mean Gaussian, with standard deviation from the literature (table 2). Third, we adjusted the neural network hyperparameters to avoid over-fitting. This includes (i) reducing network complexity and total epochs, (ii) decreasing the value of the penalty for the input reconstruction loss, (iii) adding $\ell_{2}$ weight decay regularization. Similar to §3c, a total of 54 000 input–output pairs are numerically generated according to the new prior. Of these, 4000 will be used for offline validation, and the rest is divided into the training and online testing set with a 3-to-1 ratio.

For the EHR dataset, we slightly modify the notation, where the clinical data for the q th patient (1≤q≤84) is denoted by $y^{\text{EHR},(q)}$ . A diagram for the inference task where $N_{w}$ latent variable samples are used for each EHR measurement is illustrated in equation (3.3) for the CVSim-6 model with input dimension dim⁡(v)=23 and output dimension dim⁡(y)=16 .


(3.3) $$y^{\text{EHR},(q)}\;\underset{\text{Algorithm \textasciitilde{} 1}}{\to }\;{\hat{V}}^{\text{EHR},(q)}\in R^{N_{w}\times 23}\;\underset{NN_{e}}{\to }\;{\hat{Y}}^{\text{EHR},(q)}\in R^{N_{w}\times 16}\;.$$

**Figure 12.**
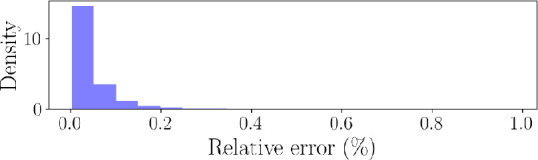
$l_{2}$ relative error distribution for the emulator. The maximum error on 4000 validation examples is equal to 0.984%.

Note that the emulator $NN_{e}$ is employed to replace the exact CVSim-6 simulator during the output reconstruction process from ${\hat{V}}^{\text{EHR}}$ to ${\hat{Y}}^{\text{EHR}}$ . There are two reasons for this. First, the emulator is significantly faster. The speedup is approximately 240 times for a single CVSim-6 system evaluation, and the cost of training the emulator is approximately 3.8 h on a home desktop with Ryzen-9 5900X CPU and 32 GB of memory. For more training details, please refer to appendix B.2. Second, the exact numerical integrator may crash due to an input parameter combination resulting from an extremely poor inversion task, while the network model can still make a prediction without causing the code to terminate. Moreover, to further justify the use of an emulator, the histogram for the $l_{2}$ norm of the prediction error on the validation dataset of size 4000 is shown in figure 12.

#### (i) Inversion results

In this section, we quantify how much noise should be added to the CVSim-6 outputs such that an inVAErt network could achieve sufficient generalization on real data in general, and our EHR dataset in particular. To do so, we introduce a scale factor δ which amplifies the values of standard deviation in table 2. This parameter quantifies the amount of noise added during training where, for example, δ=0.0 corresponds to no noise added, and δ=2.0 is used to denote a zero-mean Gaussian noise with standard deviation twice the value listed in table 2.

In addition, an error criterion to quantify the accuracy of the inversion task on a per-output component basis is defined in equation (3.4):


(3.4) $$\begin{array}{lcr} & e_{k}=\frac{1}{|P_{k}|}\cdot \frac{1}{N_{w}}\sum_{q\in P_{k}}\sum_{i=1}^{N_{w}}|y_{k}^{\text{EHR},(q)}-{\hat{Y}}_{ik}^{\text{EHR},(q)}|,\;k=1:16, & \end{array}$$

where $P_{k}$ contains the identities of the patients whose k th attribute is not missing, and $|P_{k}|$ is the cardinality of this set, listed under the ‘counts’ column in table 5. Inversion is performed with four noise levels ( δ=0.25,0.5,1.0,2.0 ), with results listed in table 5. The lowest errors are obtained for δ=0.5 . Note that we use four networks with the same hyperparameters, one for each noise intensity (§B.2), even though improved results may be obtained by fine-tuning hyperparameters on training dataset for each noise level. In addition, note that inversion is only performed for patients having more than 10 measurements, that is, 46 out of 84 patients. Also note that, generally speaking, the fewer missing components, the harder the inversion task becomes. This is because EHR measurements usually fall on the tail of the distribution learned from the synthetic CVSim-6 outputs, due to model misspecification and noise corruption.

**Table 5.** Reconstruction error per output component ( $N_{w}=100$ ) for the EHR missing data inversion, using the criterion defined in (3.3), for noise levels: δ=0.25,0.5,1.0,2.0 . The minimum error across all four cases is highlighted using bold fonts. Only patients with more than 10 available measurements (i.e. 46 out of 84) are included. The total number of patients for which a given attribute was measured is also reported in the last column. Finally, the left ventricular systolic volume $V_{l,sys}$ is never measured in the selected patients.

num.	quantity	δ=0.25	δ=0.5	δ=1.0	δ=2.0	unit	counts
1.	Hr	0.602	0.440	0.843	2.636	(bpm)	46/46
2.	$P_{a,sys}$	5.204	2.284	3.379	2.738	(mm Hg)	46/46
3.	$P_{a,dia}$	3.614	1.605	2.045	2.627	(mm Hg)	46/46
4.	$P_{r,sys}$	1.669	1.323	1.469	1.476	(mm Hg)	44/46
5.	$P_{r,dia}$	1.170	1.684	2.022	3.383	(mm Hg)	11/46
6.	$P_{pa,sys}$	1.907	1.227	1.606	1.386	(mm Hg)	46/46
7.	$P_{pa,dia}$	1.285	1.062	1.065	1.290	(mm Hg)	46/46
8.	$P_{r,edp}$	0.540	0.599	0.786	2.787	(mm Hg)	44/46
9.	$P_{w}$	0.887	0.740	1.258	1.200	(mm Hg)	46/46
10.	$P_{cvp}$	1.194	0.371	0.733	0.821	(mm Hg)	3/46
11.	$V_{l,sys}$	None	None	None	None	(ml)	0/46
12.	$V_{l,dia}$	50.960	47.391	103.402	119.440	(ml)	3/46
13.	LVEF	0.012	0.008	0.015	0.024	(−)	45/46
14.	CO	0.200	0.121	0.281	0.274	(l min^−1^)	46/46
15.	SVR	28.755	24.728	25.896	41.233	(dyn s cm^−^ ${,}^{5}$ )	46/46
16.	PVR	1.924	1.812	2.010	3.795	(dyn s cm^−^ ${,}^{5}$ )	46/46

**Figure 13.**
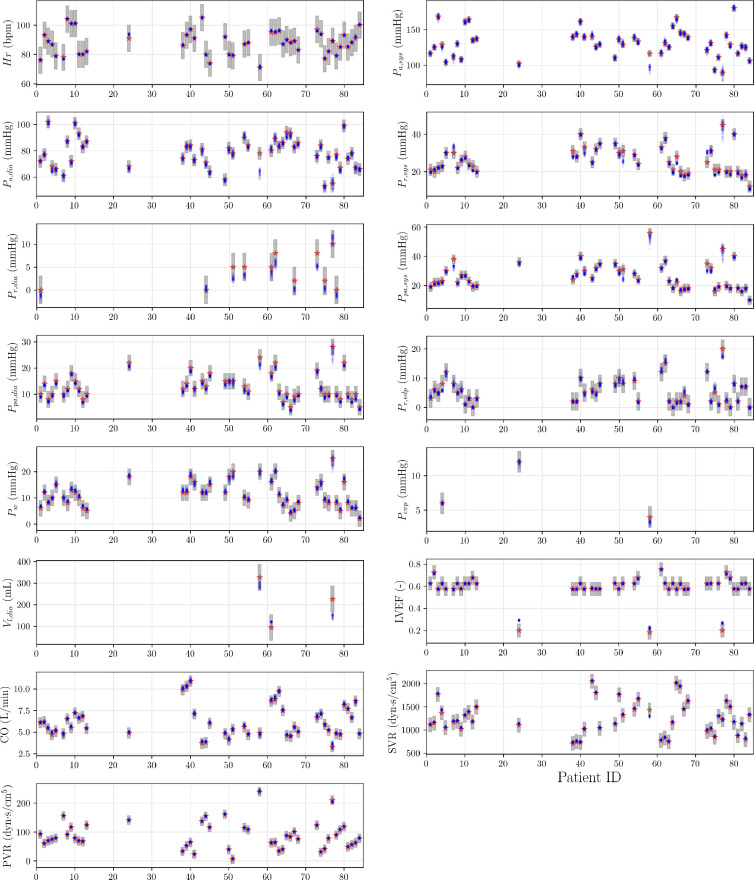
Patient-specific predictions and uncertainty quantification for each CVSim-6 output component when the noise intensity factor is set as δ=0.5 . The red star is the EHR measurement of each patient and the blue dots are the network predictions, with each dot associated with a different latent variable realization. The grey interval is obtained from the EHR measurement ±3σ , using the standard deviations listed in table 2. The left ventricular systolic volume, $V_{l,sys}$ , is missing for all patients with more than 10 measurements, hence the corresponding plot is omitted.

The patient-specific prediction is validated with respect to each of the CVSim-6 output attributes and shown in figure 13, with respect to the noise level δ=0.5 . A bounding interval of each EHR measurement ±3σ , using the reported uncertainty listed in table 2, is also superimposed to quantify the prediction accuracy. From figure 13, we notice most of the input predictions can lead to an output close to the corresponding EHR measurement, although inaccuracy and large uncertainty exist in a few cases. Among all the output targets, the arterial systolic pressure, i.e. $P_{a,sys}$ , is the most uncertain attribute in our experiment, despite when δ=0.5 , the lowest prediction error is obtained.

## 4. Discussion and conclusions

In this article, we propose a new model synthesis approach for stiff dynamical systems and demonstrate its potential using both synthetic and real data with missing components from an EHR dataset. Once an inVAErt network is trained, it can be used as an *enhanced digital twin* that can efficiently (i.e. at the cost of a single network evaluation) perform a number of complementary tasks. These include interrogation of the forward map through a fast neural emulator, physics-based likelihood evaluation and generation of new outputs compatible with a given *a priori* selected range of input parameters, and the amortized solution of an inverse problem from vectors of observations, possibly containing missing components. In addition, a unique feature of inVAErt networks is their ability to solve ill-posed inverse problems with multiple solutions, by computing an entire manifold of parameters for which model outputs correspond to the same clinical observation. We believe that a paradigm where *all* the solutions of an inverse problem are provided to the analyst (with an accuracy that only depends on the amount of training data provided to the system) is superior to any form of regularization.

Moreover, besides using a latent space to handle structural non-identifiability, for the first time, noise from a closed-form model is added during the training of an inVAErt network to tackle practical non-identifiability in the solution of inverse problems. We focus on an application in computational physiology, using the CVSim-6 six-compartment lumped‐parameter model. We analyse this system in detail, showing that its stiffness is due to the presence of ideal unidirectional valves modelled through indicator functions, and ventricular–arterial coupling during late systole inducing very small RC constants. InVAErt successfully leverages this stiff system in the presence of structural non-identifiability due to model overparameterization, and practical non-identifiability induced by noise corruption, missing data and misspecified physics. We also utilize parallel charts to represent the high-dimensional, non-identifiable manifold, facilitating the visualization of parameter variation, correlation and identifiability. A linear dimensionality reduction study of the resulting high-dimensional manifold also suggests the potential of model reparameterization, which would improve the simulation efficiency and enhance parameter identifiability.

There are many possible directions for future work, such as focusing on applications involving higher‐fidelity models such as one- or three-dimensional cardiovascular models, a broader family of latent space distributions, misspecified models and beyond.

## Figures and Tables

**Figure 14.**
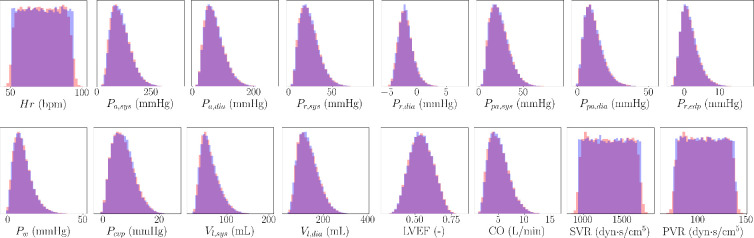
Marginal density estimation of the CVSim-6 system outputs. Blue: exact data distribution. Red: predicted distribution via the trained Real-NVP density estimator $NN_{f}$ .

**Figure 15.**
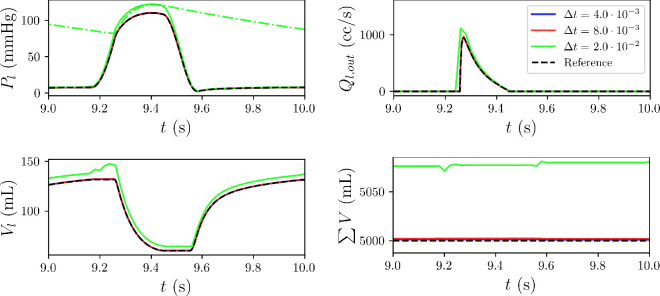
Comparison between numerical approximations for left ventricular pressure ( $P_{l}$ ), outflow ( $Q_{l,out}$ ), volume ( $V_{l}$ ) and total stressed volume ( ∑V ). We compare an implicit fourth-order Runge–Kutta method with three different time‐step sizes equal to $\Delta t=4\times 10^{-3}$ (s), $8\times 10^{-3}$ (s) and $2\times 10^{-2}$ (s). The reference approach is an implicit fifth-order Runge–Kutta method (Radau [13]) with adaptive step size. The systemic arterial pressure solution $P_{a}$ when $\Delta t=2\times 10^{-2}$ (s) is also super-imposed in the top-left figure as the green dash-dotted line.

## Data Availability

Our code and data are provided in a github repository: https://github.com/desResLab/In-VAErt4Cardio.

## Appendix A. Additional details on the CVSim-6 model formulation

The following physiological quantities can be computed from the input parameters listed in table 1:

$$\begin{array}{r} \left\{ \begin{array}{ll} \text{Total unstressed blood volume:}\; & V_{tot}^{0}=V_{l}^{0}+V_{a}^{0}+V_{v}^{0}+V_{r}^{0}+V_{pa}^{0}+V_{pv}^{0}\;\text{(mL)}\;,\\ \text{Duration of cardiac cycle:}\; & T_{tot}=60/Hr\;\text{(s)}\;,\\ \text{Systolic time per cardiac cycle:}\; & T_{sys}=T_{tot}\cdot r_{sys}\;\text{(s)}\;,\\ \text{Diastolic time per cardiac cycle:}\; & T_{dia}=T_{tot}-T_{sys}\;\text{(s)}\;, \end{array}\right. \end{array}$$

and we assume a constant total blood volume $V_{tot}=5000$ (ml), representative of an adult male weighing 70 kg [6]. To begin the *in silico* simulation of the CVSim-6 system and preserve mass conservation at t=0 , the initial pressures in the ODE system equation (2.1) are determined by solving the following set of equations [6]

(A 1)
$$\begin{array}{r} \left\{ \begin{array}{ll} C_{l,dia}(P_{l,dia}^{0}-P_{th}) & -\;C_{l,sys}(P_{l,sys}^{0}-P_{th})\\ & =C_{r,dia}(P_{r,dia}^{0}-P_{th})-C_{r,sys}(P_{r,sys}^{0}-P_{th})\;,\\ & =T_{sys}\frac{P_{l,sys}^{0}-P_{a}^{0}}{R_{l,out}}\;,\\ & =T_{tot}\frac{P_{a}^{0}-P_{v}^{0}}{R_{a}}\;,\\ & =T_{dia}\frac{P_{v}^{0}-P_{r,dia}^{0}}{R_{r,in}}\;,\\ & =T_{sys}\frac{P_{r,sys}^{0}-P_{pa}^{0}}{R_{r,out}}\;,\\ & =T_{tot}\frac{P_{pa}^{0}-P_{pv}^{0}}{R_{pv}}\;,\\ & =T_{dia}\frac{P_{pv}^{0}-P_{l,dia}^{0}}{R_{l,in}}\;,\\ V_{tot}-V_{tot}^{0} & =C_{l,dia}(P_{l,dia}^{0}-P_{th})+C_{a}(P_{a}^{0}-\frac{1}{3}P_{th})+C_{v}P_{v}^{0}\\ & +\;C_{r,dia}(P_{r,dia}^{0}-P_{th})+C_{pa}(P_{pa}^{0}-P_{th})+C_{pv}(P_{pv}^{0}-P_{th})\;, \end{array}\right. \end{array}$$

where $P_{\left(\cdot \right)}^{0}=P_{\left(\cdot \right)}(t=0)$ and the ventricular pressures are initialized at their diastolic values, namely,

$$P_{l}(t=0)=P_{l,dia}^{0},\;P_{r}(t=0)=P_{r,dia}^{0}.$$

The time-varying capacitance of the two ventricles utilized in equation (2.1) are defined through their reciprocals, i.e. through the elastance,

$$E_{l}(t)=1/C_{l}(t),\;E_{r}(t)=1/C_{r}(t),$$

via the following *activation functions* [6,26] (these formulae are obtained from CVSim, an open-source code repository within the PhysioNet project [47]):

(A 2)
$$\begin{array}{rl} E_{l}(t) & =\left\{ \begin{array}{ll} \frac{1}{C_{l,dia}}, & \;t=0\;\text{or}\;t>1.5\cdot T_{sys},\\ \frac{1}{2}(\frac{1}{C_{l,sys}}-\frac{1}{C_{l,dia}})(1-\cos(\pi \frac{t}{T_{sys}}))+\frac{1}{C_{l,dia}}, & \;0<t\leq T_{sys},\\ \frac{1}{2}(\frac{1}{C_{l,sys}}-\frac{1}{C_{l,dia}})(1+\cos(2\pi \frac{t-T_{sys}}{T_{sys}}))+\frac{1}{C_{l,dia}}, & \;T_{sys}<t\leq 1.5\cdot T_{sys}. \end{array}\right.\\ E_{r}(t) & =\left\{ \begin{array}{ll} \frac{1}{C_{r,dia}}, & \;t=0\;\text{or}\;t>1.5\cdot T_{sys},\\ \frac{1}{2}(\frac{1}{C_{r,sys}}-\frac{1}{C_{r,dia}})(1-\cos(\pi \frac{t}{T_{sys}}))+\frac{1}{C_{r,dia}}, & \;0<t\leq T_{sys},\\ \frac{1}{2}(\frac{1}{C_{r,sys}}-\frac{1}{C_{r,dia}})(1+\cos(2\pi \frac{t-T_{sys}}{T_{sys}}))+\frac{1}{C_{r,dia}}, & \;T_{sys}<t\leq 1.5\cdot T_{sys}. \end{array}\right. \end{array}$$

At each step, the stressed volume at each compartment is calculated by the linear pressure–volume relationship [6]

(A 3)
$$\begin{array}{r} \left\{ \begin{array}{l} V_{l}(t)=V_{l}^{0}+(P_{l}(t)-P_{th})C_{l}(t),\\ V_{a}(t)=V_{a}^{0}+(P_{a}(t)-\frac{1}{3}P_{th})C_{a},\\ V_{v}(t)=V_{v}^{0}+P_{v}(t)C_{v},\\ V_{r}(t)=V_{r}^{0}+(P_{r}(t)-P_{th})C_{r}(t),\\ V_{pa}(t)=V_{pa}^{0}+(P_{pa}(t)-P_{th})C_{pa},\\ V_{pv}(t)=V_{pv}^{0}+(P_{pv}(t)-P_{th})C_{pv}, \end{array}\right. \end{array}$$

and their sum: $\sum V(t)=V_{l}(t)+V_{a}(t)+V_{v}(t)+V_{r}(t)+V_{pa}(t)+V_{pv}(t)$ at any given time t should be consistent with the previously specified constant $V_{tot}=5000$ (ml). This constraint provides a practical way to validate the numerical solver for the CVSim-6 system, as demonstrated in figure 7.

## Appendix B. Details of neural network modelling

We build and train inVAErt networks using the PyTorch open-source machine learning platform [48], together with an Adam optimizer [49] and a StepLR learning rate scheduler. The fully connected MLP networks are the building blocks of the neural emulator $NN_{e}$ , variational encoder $NN_{v}$ and the decoder $NN_{d}$ . As for the density estimator $NN_{f}$ , we implement the Real-NVP normalizing flow architecture [29,50]. For the network activations, except for the density estimator, where the recommended functions in [29] are adopted, we use the smoothed ReLU function *Swish* [51] due to its superior performance over other choices.

Furthermore, the dimension of the latent space is initially set as the difference between the input and output dimensionality, i.e. dim⁡(w)=dim⁡(v)−dim⁡(y) . As a result of extensive testing from a previous investigation [17], we found that an increase in the latent space dimensionality with respect to the above difference can improve learning of complex non-identifiable manifolds. Therefore, we consider the extra dimensionality as a hyperparameter and select it via grid search or optimization. Regarding the selection of penalty parameters during training, we follow the rule where $\beta_{d}\gg \beta_{v},\beta_{r}$ , which proves to be effective in reducing the occurrence of posterior collapse, a common issue in VAE-based models. For additional information about inVAErt network training, interested readers may refer to [17]. Several latent space sampling strategies were also discussed in [17] to improve accuracy of the inverse predictions. In this article, the PC-sampling (predictor–corrector) scheme with two iterations is implemented as a default setting.

### B.1. Study of structural non-identifiability

In this section, we provide hyper-parameter details of the network model utilized in §3c please see (tables 6 and 7).

**Table 6.** Summary of InVAErt modules for the CVSim-6 system with synthetic data.

module	hidden units	num. of layers	activation	total parameters
$NN_{e}$	60	8	SiLU	24 376
$NN_{v}$	32	6	SiLU	6246
$NN_{d}$	64	6	SiLU	20 439

module	hidden units	layers per block	blocks	activation	batchNorm	total parameters
$NN_{f}$	24	4	16	Tanh, ReLU	False	51 712

**Table 7.** Hyperparameter choices for the network training: CVSim-6 system with synthetic data. The penalties for the loss functions are $[\beta_{v},\beta_{d},\beta_{r}]=\left[1,2000,20\right]$ and the latent space dimensionality is dim⁡(w)=19 .

module	minibatch size	initial lr	total epochs	epochs per decay	decay rate
$NN_{e}$	256	$1\times 10^{-3}$	20 000	100	0.98
$NN_{f}$	512	$2\times 10^{-3}$	1500	200	0.85
$NN_{v}+NN_{d}$	256	$1\times 10^{-3}$	20 000	100	0.985

### B.2. Study of the EHR dataset

In this section, we provide hyper-parameter details of the network models and hyperparameters utilized in §3d please see (tables 8 and 9).

**Table 8.** Summary of InVAErt modules for the study of the EHR dataset.

module	hidden units	num. of layers	activation	total parameters
$NN_{e}$	80	8	SiLU	42 096
$NN_{v}$	32	6	SiLU	6246
$NN_{d}$	64	6	SiLU	20 439

module	hidden units	layers per block	blocks	activation	batchNorm	total parameters
$NN_{f}$	18	4	10	Tanh, ReLU	True	20 280

**Table 9.** Hyperparameter choices for the network training: CVSim-6 system with the EHR dataset. In the training of density estimator $NN_{f}$ and the inverse model $NN_{v}+NN_{d}$ , $l_{2}$ weight decay is implemented with a penalty of $2\times 10^{-4}$ . The penalties for the loss functions are $\left[\beta_{v},\beta_{d},\beta_{r}]=[1,1000,10\right]$ and the latent space dimensionality is dim⁡(w)=19 .

module	minibatch size	initial lr	total Epochs	epochs per decay	decay rate
$NN_{e}$	256	$3\times 10^{-3}$	25 000	100	0.98
$NN_{f}$	512	$4\times 10^{-3}$	1000	200	0.5
$NN_{v}+NN_{d}$	512	$1\times 10^{-3}$	2000	100	0.97

## Appendix C. Additional results

### C.1. Forward emulation and density estimation

In this section, we evaluate the performance of InVAErt components $NN_{e}$ and $NN_{f}$ for the CVSim-6 system studied in §3c. First, we test the trained neural emulator $NN_{e}$ via the validation dataset, and summarize the absolute prediction error for each output component in table 10, where a noticeable overall accuracy can be found.

**Table 10.** Statistics of the emulator prediction error across the validation dataset of size 4000.

	Hr (bpm)	$P_{a,sys}$ (mm Hg)	$P_{a,dia}$ (mm Hg)	$P_{r,sys}$ (mm Hg)	$P_{r,dia}$ (mm Hg)
average	9.81 × 10^−3^	4.72 × 10^−2^	4.52 × 10^−2^	1.20 × 10^−2^	3.44 × 10^−2^
max	7.57 × 10^−2^	6.68 × 10^−1^	3.92 × 10^−1^	2.35 × 10^−1^	2.33 × 10^−2^
std	8.59 × 10^−3^	4.65 × 10^−2^	4.02 × 10^−2^	1.26 × 10^−2^	2.81 × 10^−3^

	$P_{pa,sys}$ (ml)	$P_{pa,dia}$ (mm Hg)	$P_{r,edp}$ (mm Hg)	$P_{w}$ (mm Hg)	$P_{cvp}$ (mm Hg)
average	1.10 × 10^−2^	9.77 × 10^−3^	1.08 × 10^−4^	1.91 × 10^−3^	3.68 × 10^−3^
max	2.87 × 10^−1^	1.86 × 10^−1^	2.04 × 10^−3^	2.00 × 10^−2^	4.24 × 10^−2^
std	1.18 × 10^−2^	9.94 × 10^−3^	1.01 × 10^−4^	1.82 × 10^−3^	3.49 × 10^−3^

	$V_{l,sys}$ (mm Hg)	$V_{l,dia}$ (ml)	LVEF (−)	CO (l min^−1^)	SVR (dyn s cm^−5^)	PVR (dyn s cm^−5^)
average	2.33 × 10^−2^	4.45 × 10^−2^	2.74 × 10^−3^	7.59 × 10^−3^	1.81 × 10^−1^	1.95 × 10^−2^
max	2.88 × 10^−1^	4.51 × 10^−1^	7.82 × 10^−2^	7.38 × 10^−2^	1.62 × 10^+0^	2.12 × 10^−1^
std	2.38 × 10^−2^	4.33 × 10^−2^	3.34 × 10^−2^	7.30 × 10^−3^	1.60 × 10^−1^	1.84 × 10^−2^

Next, figure 14 compares the marginal distributions estimated by the trained normalizing flow model $NN_{f}$ (red), with the exact data distributions (blue), for each output component of the CVSim-6 system. Overall, the alignment is close, despite some predicted quantity, such as Hr , exhibits a slightly heavier tail.

## Appendix D. Additional discussions on non-identifiable manifold

In §3c(ii), one specific checkpoint, i.e. table 4, was used to investigate the structural non-identifiability of the CVSim-6 system. Here, we take the entire validation set of size 4000 and analyse the variability of the input parameter predictions.

For each of the 4000 validation labels, i.e. $y^{*}$ , we sample 1000 latent variables of w from the standard Gaussian latent space and solve the inverse problems. Then, we compute the coefficient of variation (std/mean) of each input component with respect to the 1000 predictions, take the average regarding the whole validation set and list the results in table 11.

**Table 11.** Average coefficients of variation of the CVSim-6 input parameters, obtained by solving inverse problems with respect to the validation dataset of size 4000.

	Hr	$P_{th}$	$r_{sys}$	$C_{l,dia}$	$C_{l,sys}$	$C_{a}$	$C_{v}$	$C_{r,dia}$	$C_{r,sys}$	$C_{pa}$	$C_{pv}$	$R_{l,in}$
CoV (%)	0.056	10.356	6.114	4.550	6.750	1.716	24.831	2.252	21.781	3.954	24.246	17.058

	$R_{l,out}$	$R_{a}$	$R_{r,in}$	$R_{r,out}$	$R_{pv}$	$V_{l}^{0}$	$V_{a}^{0}$	$V_{v}^{0}$	$V_{r}^{0}$	$V_{pa}^{0}$	$V_{pv}^{0}$
CoV (%)	17.163	0.066	4.261	3.048	0.097	16.423	16.518	8.324	17.180	17.084	16.791

Although different output $y^{*}$ leads to distinct input parameter variability due to the nonlinear system response, the population statistics listed in table 11 are consistent with the variabilities shown in figure 9. In addition, the parameters $R_{a}$ and $R_{pv}$ exhibit consistently small coefficients of variation (i.e. figure 9), suggesting a high degree of identifiability (the heart rate Hr was copied from the inputs to the outputs, so its small variability is expected).

## Appendix E. Additional stiffness analysis

As shown in figure 7, the explicit Runge–Kutta scheme exhibits numerical instability when the time discretization is too coarse. For comparison, in this section, we utilize an implicit Runge–Kutta method with fixed time‐step sizes to integrate the CVSim-6 system. To preserve the fourth-order accuracy as its explicit counterpart, the Crouzeix’s three-stage, fourth-order diagonally Runge–Kutta method [52,53] is selected.

Comparing figure 15 to figure 7, the pressure and flow approximations are seen to be more stable and realistic with a large step size Δt . However, an explicit scheme appears to lead to better conservation of the total blood volume. We attribute this to the fact that a nonlinear system of equations needs to be solved at each time step for implicit schemes, and so the convergence of the optimization task, and the choice for the initial solution guess, may negatively affect volume conservation. In this context, we find that the step size has to be smaller than $2\times 10^{-3}$ s for a scheme with constant step size to produce results comparable to an adaptive scheme. This step size is also consistent with the smallest RC timescale constant computed in §3a.
